# *α*-D-Glucose as a non-radioactive MRS tracer for metabolic studies of the brain

**DOI:** 10.1038/s41598-023-33161-8

**Published:** 2023-04-15

**Authors:** Guodong Weng, Piotr Radojewski, Johannes Slotboom

**Affiliations:** 1 Institute for Diagnostic and Interventional Neuroradiology, Support Center for Advanced Neuroimaging (SCAN), Bern University Hospital, Inselspital, University of Bern, Bern, Switzerland; 2 Translational Imaging Center, Sitem-Insel, Bern, Switzerland

**Keywords:** Blood-brain barrier, Metabolism, Magnetic resonance imaging, Monosaccharides, Translational research

## Abstract

Changes in brain glucose metabolism occur in many neurological disorders as well as during aging. Most studies on the uptake of glucose in the brain use positron emission tomography, which requires injection of a radioactive tracer. Our study shows that ultra-high-field ^1^H-MRS can be used to measure α-d-glucose at 5.22 ppm in vivo, and the α-d-glucose can be used as a radiation-free tracer in the human brain.

## Introduction

Glucose, C_6_H_12_O_6_, is the most abundant carbohydrate in nature. Living organisms can metabolize glucose to CO_2_ and H_2_O enabling them to produce ATP via anaerobic or aerobic glycolysis. Due to its central role in metabolism, in vivo investigations of glucose are of the utmost interest. Currently, most studies of brain metabolism in vivo use positron emission tomography (PET) with ^18^F-fluorodeoxyglucose. A major drawback of PET is the exposure to ionizing radiation. Due to its non-invasive nature, magnetic resonance spectroscopy (MRS) has also been used to measure glucose in vivo in numerous studies. Most of these studies were performed using ^13^C-MRS^1^, and more recently, ^2^H-MRS despite their low sensitivity and low spatial resolution^2^. ^1^H-MRS has higher sensitivity and is the MRS technology most widely used in studies of the brain. However, ^1^H-MRS is not widely used in in vivo metabolic studies of glucose for three reasons: First, the upfield glucose spectrum overlaps with that of other metabolites, making accurate glucose quantification challenging. Second, although the downfield glucose signal consists of an isolated doublet at 5.22 ppm, this is very close to the water resonance at 4.65 ppm, requiring good water suppression. Third, the proton spins of glucose are J-coupled and undergo J-evolution, which further decreases their signal intensity.

Recently, a novel spectral editing technique, SLOW-editing, was designed for 3D-spatial-resolved whole brain ^1^H-MRS measurements at ultra-high magnetic field (UHF)^3^. We propose to use the SLOW-editing sequence for the whole brain detection of the natural downfield *α-d-*glucose signal at 5.22 ppm and to study the changes of the *α-d-*glucose signal at 5.22 ppm before and after oral ingestion of an *α-d-*glucose solution.

**Figure 1:**
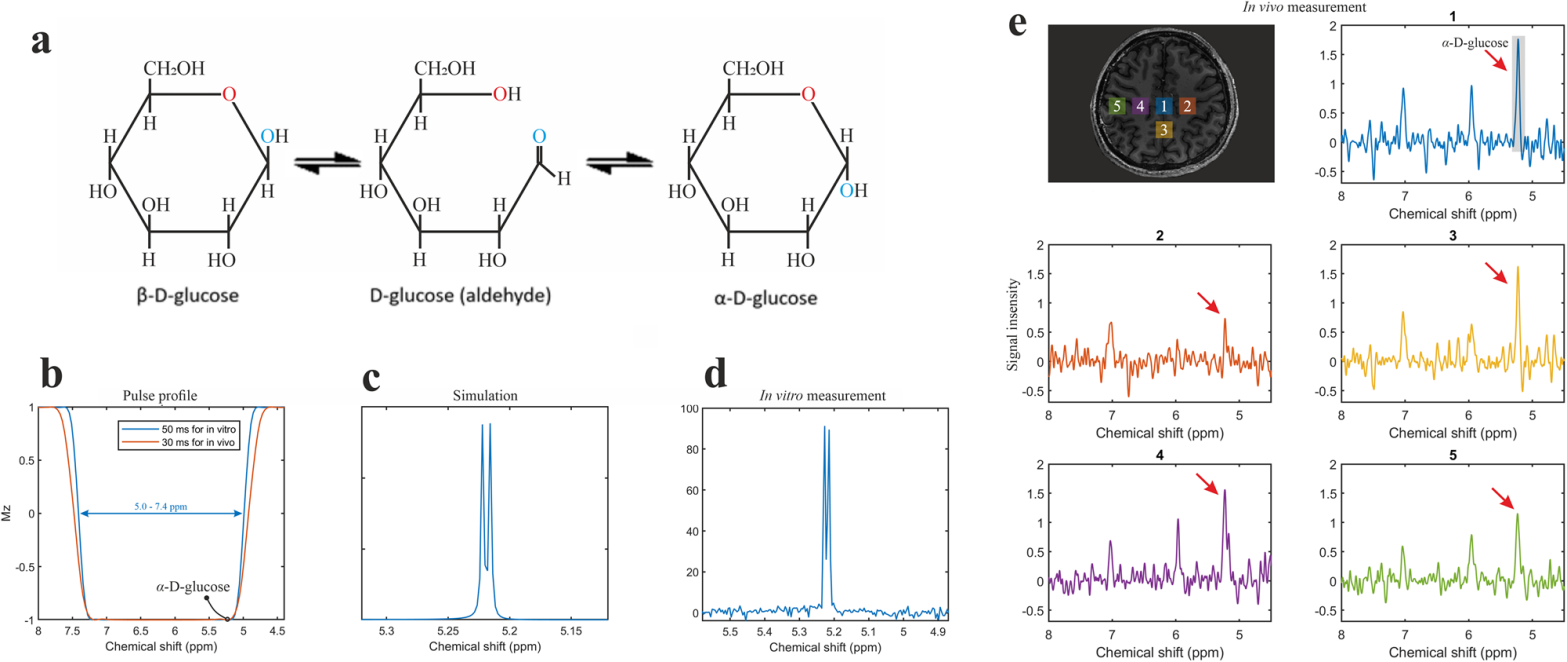
(**a**) d-glucose has three isomers: two closed ring structures (*α-d-* and *β-d-*glucose) and one linear form (aldehyde). The two cyclic isomers have slightly different physical properties resulting in different NMR-spectra. The *α-d-*glucose isomer has a unique feature in the spectrum: a doublet at 5.22 ppm, which is absent in the spectrum of *β-d-*glucose. (**b**) Pulse profiles of refocusing/editing CSAP used in vitro and in vivo measurements. (**c**) The simulation of *α-d-*glucose at 5.22 ppm. (**d**) In vitro phantom measurement (TE = 120 ms). (**e**) In vivo measurement in healthy subject 1 shows abundant *α*-d-glucose (Scheme 1, TE = 80 ms).

If pure crystalline *α-d-*glucose is dissolved in a fluid, a process of mutarotation takes place. During mutarotation the cyclic *α*-d-glucose is first converted to a linear (aldehyde) isomer, and the linear form is then converted into the second cyclic form of glucose namely *β-d-*glucose (Fig. 1a). At room temperature the mutarotation results in an equilibrium concentration ratio of *α*-d-glucose-/*β*-d-glucose of approximately 36 to 64%.

We hypothesize that a non-equilibrium α*-d-*glucose solution can be used as a tracer for UHF magnetic resonance spectroscopic imaging (MRSI). Specifically, freshly dissolved pure crystalline *α-d-*glucose solution in a non-equilibrium state can be orally administered, will be absorbed in the intestine, and transported to the organs via blood vessels.

**Figure 2:**
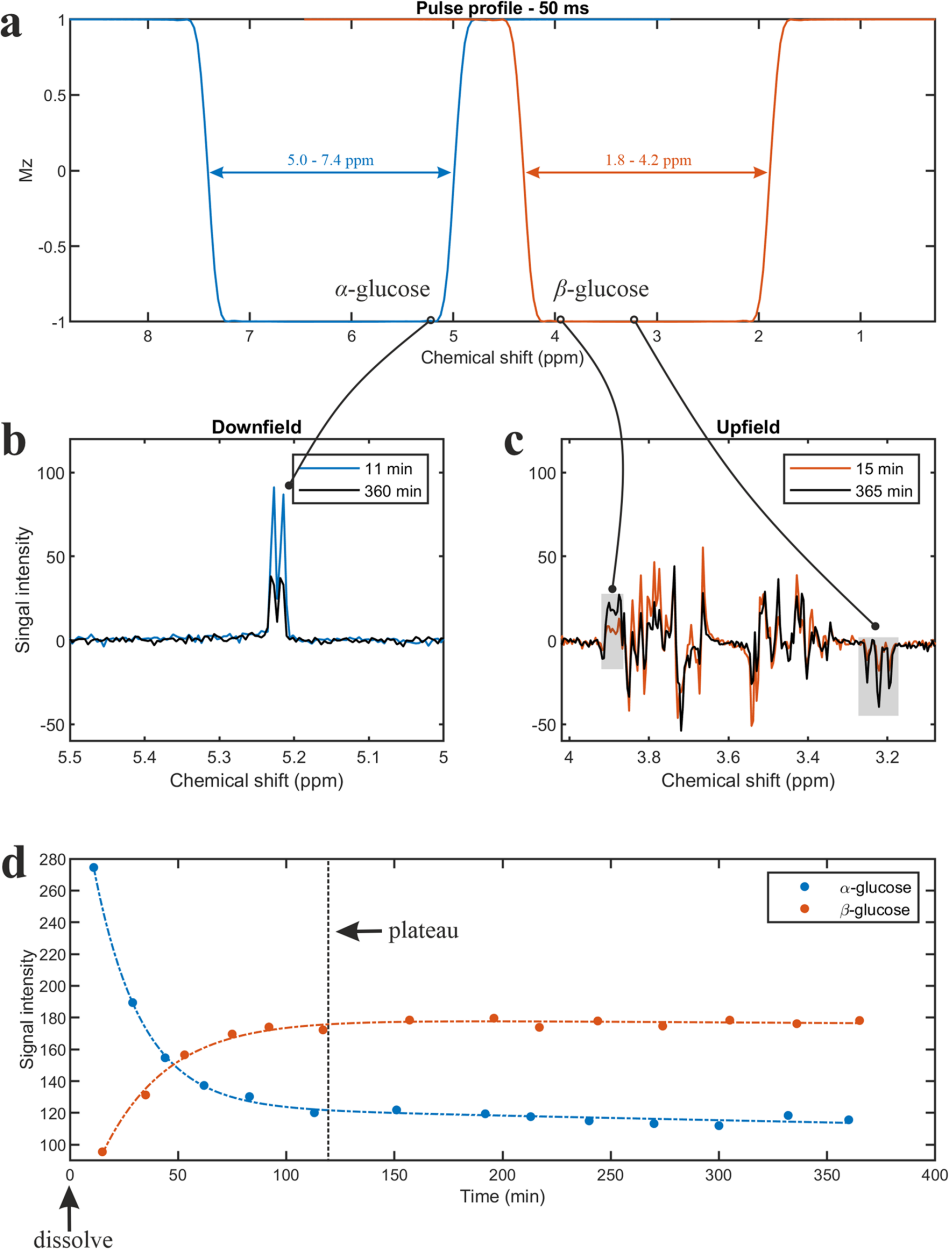
In vitro measurement using SLOW-editing. (**a**) Pulse profiles of refocusing/editing CSAP for downfield and upfield measurements. (**b**,**c**) Phantom measurements for two different time points after dissolving crystalline *α-d-*glucose. (**d**) Plot of peak integration of *α*/*β-d-*glucose at 5.22 and 3.88 ppm against time after dissolution of *α-d-*glucose.

**Figure 3:**
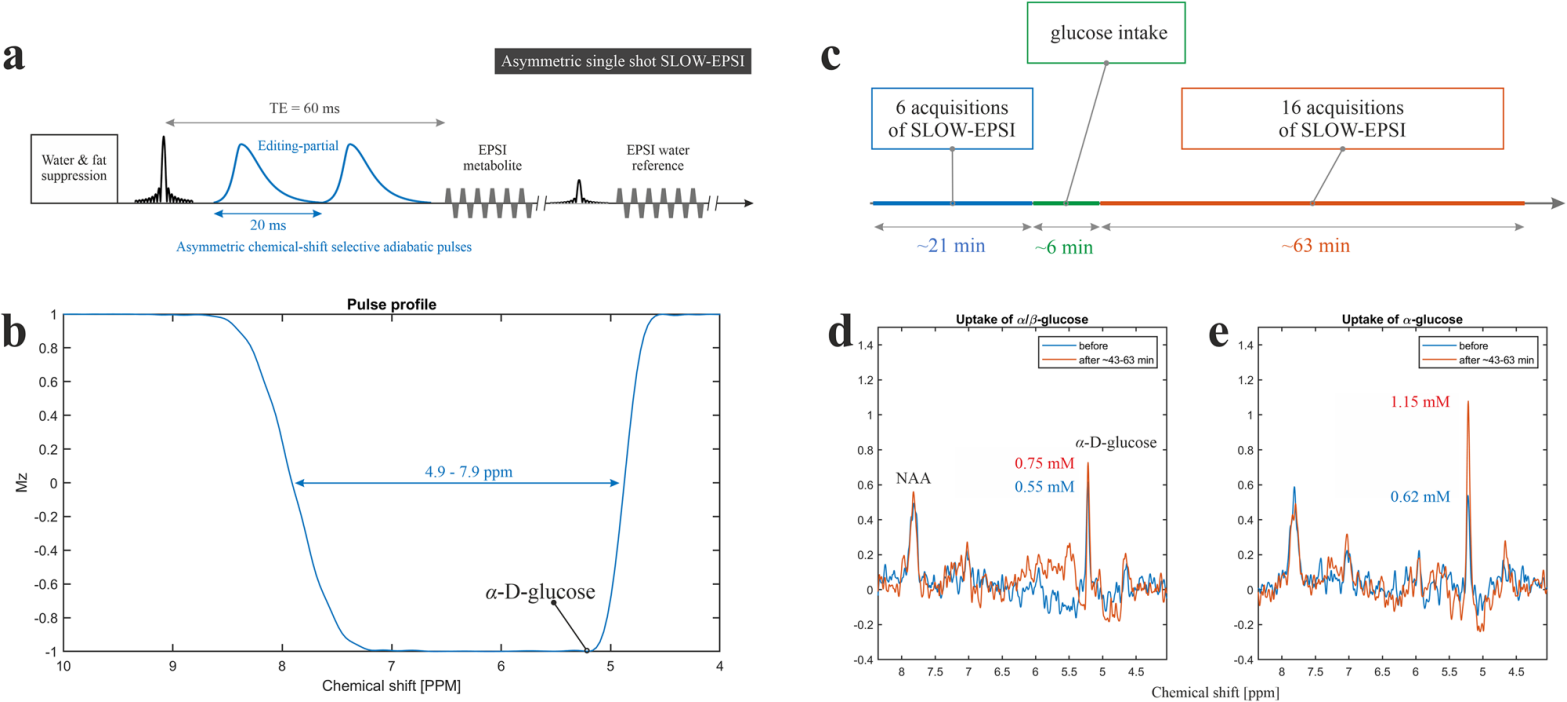
(**a**) Single-shot SLOW-editing EPSI-based sequence used (Scheme 2), which makes use of an asymmetric 2π-CSAP. (**b**) Pulse profiles of the asymmetric refocusing/editing CSAP. (**c**) Flowchart illustrating the in vivo MRSI measurements in subject 2. (**d**) The downfield spectra before (blue) and after (orange) uptake of *α/β*-equilibrium glucose. (**e**) The downfield spectra before (blue) and after (orange) uptake of *α-d-*glucose.

To test this hypothesis, we first simulated the MR spectrum of *α-d-*glucose at 5.22 ppm under SLOW-editing conditions. The corresponding in vitro phantom spectrum of *α*-d-glucose confirmed the simulated 5.22 ppm *α-d-*glucose signal (Fig. 1b–d). An in vivo MR measurement in a healthy volunteer (Subject 1) revealed an abundant *α*-d-glucose signal at 5.22 ppm (Fig. 1e). We then measured the mutarotation process in vitro as a function of time and observed the establishment of the *α/β-d-*glucose equilibrium after 120 min. The time-evolution of the *α-* and* β-d-*glucose was assessed by integration of the 5.22 and 3.88 ppm resonances and showed a reversed pattern and both reached a plateau at approximately 120 min (Fig. 2). Finally, we performed two measurements in a second healthy volunteer (Subject 2) before and after ingestion of equilibrium *α/β-d-*glucose solution as well as before and after ingestion of a non-equilibrium *α-d-*glucose solution (Fig. 3). The first measurement revealed an estimated baseline *α-d-*glucose concentration in the brain of 0.55 mM and a maximum value of 0.75 mM between ~ 43 and 63 min after intake, i.e. the *α-d-*glucose concentration increased by a factor 1.36 (Fig. 3d). At baseline, the estimated total α- and β-glucose concertation was 1.53 mM (1.53 = 0.55/36%, based on the assumption of α-glucose to β-glucose ratio in vivo of 36:64). The second measurement revealed an *α-d-*glucose estimated concentration baseline of 0.62 mM and a maximum value of 1.15 mM between ~ 43 and 63 min after intake, i.e. it increased by a factor of 1.85 (Fig. 3e). At baseline, the estimated total *α*- and *β*-glucose concertation was 1.72 mM (1.72 = 0.62/36%). After the ingestion of the equilibrium *α/β-d-*glucose we found a 36% increase in the *α-d-*glucose peak, which is in line with the 34% increase in blood glucose concentration recorded in an independent control measurement performed after ingestion of an off-the-shelf regular glucose solution (Supplementary Fig. S1). Following the ingestion of the non-equilibrium *α-d-*glucose solution, we observed a higher increase of 85%*.* This suggests that non-equilibrium *α-d-*glucose solution can cross the blood–brain barrier, and that this effect could be used to improve the sensitivity for detecting *α-d-*glucose changes in vivo in the brain.

**Scheme 1:**
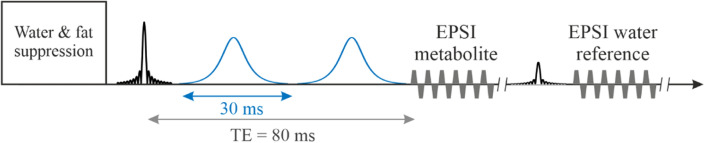
Single-shot SLOW-EPSI sequences which makes use of phase compensated adiabatic 2π-pulses with complex secant hyperbolic modulation type (so called 2π-CSAP). The pulse durations of the 2π-CSAP pulses is 30 ms for scheme 1.

**Scheme 2:**
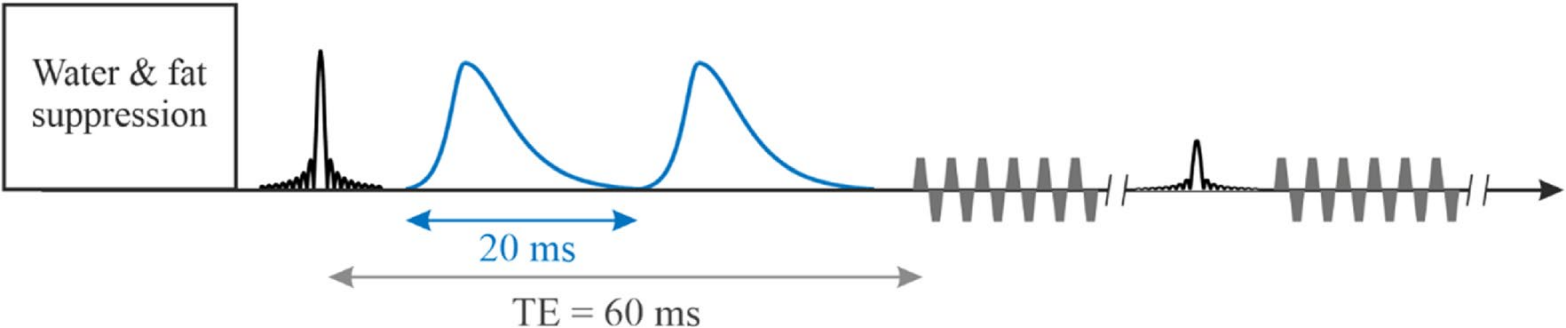
Single-shot SLOW-EPSI sequences which makes use of phase compensated adiabatic 2π-pulses with complex secant hyperbolic modulation type (so called 2π-CSAP). The pulse durations of the 2π-CSAP pulses is 20 ms for scheme 2.

Our findings demonstrate that single-shot SLOW-EPSI-based detection of the natural *α-d-*glucose signal is feasible both in vitro and in vivo with 100% editing efficiency. Moreover, the *α-d-*glucose signal increase can be measured in vivo after oral administration. The effect of mutarotation of an initially pure *α-d-*glucose solution could be measured in vitro*.* At room temperature (24 °C), the time needed to reach the equilibrium *α/β-d-*glucose solution was approximately 120 min*.* Since mutarotation is a thermodynamic effect, at body temperature (37 °C) we expect the time to reach *α*/*β-d-*glucose equilibrium to be shorter. The use of single-shot SLOW-EPSI with asymmetric radiofrequency (RF)-pulses enabled shortening of the echo time (TE) from 80 ms (Scheme 1, online “Methods” section) to 60 ms (Scheme 2, online “Methods” section), and thus increased the signal-to-noise ratio of the spectra. The *α-d-*glucose at 5.22 ppm is fully in-phase due to the spectral editing effect resulting in a signal intensity increase^3^. The excellent additional water suppression using chemical-shift selective adiabatic 2π-pulses (2π-CSAP)^3^ allows detection of the well resolved peak near the water signal (4.65 ppm), which is difficult with conventional water suppression techniques only (i.e. VAPOR and WET)^4^. One limitation of SLOW-EPSI can be lipid contamination at ~ 5.3–5.5 ppm, which can compromise the spectral quality of *α-d-*glucose. However, application of further lipid suppression techniques should address this issue^4^.

To the best of our knowledge, this is the first report demonstrating that the mutarotation effect of non-equilibrium *α/β-d-*glucose solutions can be measured in vivo in brain tissue. This principle could be utilized to conduct in vivo whole brain studies without the need for ionizing radiation. Since crystalline *α-d-*glucose costs considerably less than ^18^F-fluorodeoxyglucose, ^13^C and ^2^H, a cheaper and radiation-free tracer would be available for large scale in vivo studies of glucose metabolism in the brain. This could enable long-term longitudinal observational studies over the entire lifespan with no risk of radiation exposure.

This study has limitations that need to be considered when interpreting the results. First, like all MR-spectroscopy based methods the sensitivity is limited, especially in comparison to PET. Additionally, the 5.22 ppm doublet of *α-d-*glucose stems from a single proton, and therefore aggravates its detection. Even on a 7 T scanner the minimal concentrations that can be measured is estimated in the order of 0.1–0.2 mmol (depending on the voxel size), whereas for 19FDG-PET this is in picomolar range. Another limitation of orally administering *α-d-*glucose solution is that the *α*-d-glucose first has to pass the gastrointestinal system, making quantitative statements on the glucose transport dynamics difficult. In this respect, oral administration of deuterated glucose^2^ has the same disadvantage as the underlying method (passage of the glucose through the gastrointestinal system), but has the clear advantage that metabolic products glutamate and lactate can be obtained at the same time, allowing for more straightforward conclusions about the metabolic activity. This comes however at the price of a substantially more expensive tracer substance. Further studies should explore intravenous supplementation of *α*-d-glucose solution as well as and maintaining the blood glucose concentration on a precisely controlled level with clamp technique^5,6^.

Finally, to be discussed in the context of radiation-free imaging of glucose, is the chemical exchange saturation transfer (CEST) MRI technique. It aims at saturation of rapidly exchanging protons in molecules. Glucose CEST (or glucoCEST)^7,8^ has already shown its potential in vivo and can produce metabolite maps with high resolution. However, the proximity of the rapidly exchanging protons to the water resonance makes quantification challenging, especially at field strengths < 7 T. Due to the fact that CEST measures a proxy of the glucose present in the tissue, much relies on proper modelling, accounting for molecules in the same offset frequency range as the glucose. In contrast, the presented technique does not have this drawback, since the 5.22 ppm resonance is not overlapping with other molecules with rapidly exchanging protons.

Despite the above-described limitations, the presented study challenges the common assumptions about measuring whole-brain glucose by means of 1H-MRSI and should spark further in-depth investigations.

## Methods

All methods were carried out in accordance with relevant guidelines and regulations.

### Simulation of α-d-glucose and β-d-glucose spectra

The simulations of the spin system were performed using in-house MATLAB (R2019b) code, by solving a relaxation-free Liouville-von Neumann equation^9^.

### Non-equilibrium α-d-glucose and equilibrium α/β-d-glucose

The crystalline, water free, α*-D-*glucose used was supplied by Thermo Fisher Scientific, Inc. (Belgium; article number 10032070). The product is intended for laboratory use and was therefore toxicologically tested by UFAG Laboratorien (Sursee, Switzerland) specialized in testing of food.

For in vitro phantom measurements, the *α-D-*glucose (~ 1.8 g) was dissolved in 500 ml water and the solution was immediately filled into a spherical phantom, which was then placed in the scanner. The first SLOW measurement was started approximately 10 min after dissolving the glucose.

For in vivo measurements, the non-equilibrium *α-D-*glucose solution was obtained by dissolving (~ 70 g) in 500 ml tap water and administered orally during ~ 6 min.

The equilibrium *α*/*β-D-*glucose solution was prepared by dissolving ~ 70 g *α-D-*glucose in 500 ml tap water 650 min before the start of the in vivo measurements.

### Scanner

All MRI and MRSI acquisitions were performed on a Siemens 7 T MR scanner in clinical mode (MAGNETOM Terra, Germany) using the Nova 1Tx 32Rx head coil.

### MR-sequences

#### SLOW-EPSI

Single-shot SLOW-editing was used for all spectroscopic recordings. The sequence is based on previous work on SLOW-EPSI^3,10^. In SLOW-editing, the 2π-CSAP simultaneously acts as both refocusing and editing pulses. Conventional two-shot SLOW-editing requires two measurements: in the first measurement all spins of a J-coupled spin system are refocused, whereas in the second measurement at least one resonance of the J-coupled spin system is not refocused. The resulting “edited” spectrum is obtained by subtracting the second response (denoted as editing-partial) from the first (denoted as editing-full). Signals of all uncoupled spins are eliminated from the difference spectrum. The refocusing/editing 2π-CSAP for SLOW-editing may take 1/2 J milliseconds, which is the characteristic time needed for a J-coupled spin system to evolve into a state in which antiphase terms are present at the highest intensity.

#### Single-shot SLOW-EPSI of α-d-glucose

Since no other metabolite overlaps with α-glucose at 5.22 ppm, only the editing-partial measurement is needed, and there is no need for a second acquisition editing-full measurement followed by signal subtraction^3,11^ of the two signals. In the editing-partial response, both chemical shift and J-coupling are refocused. The bandwidth of single-shot editing ranges from 5.0–7.4 ppm (see Fig. 2a).

#### In vitro α-d-glucose using single-shot SLOW-EPSI

A phantom measurement was performed on a fresh pure *α*-d-glucose solution to evaluate the time-dependent mutarotation process towards reaching the *α/β-D-*glucose equilibrium in vitro. The phantom measurement was performed on a spherical glucose phantom prepared in-house (~ 20 mmol/L of α-d-glucose solution at a constant room temperature of 24 °C). In order to follow the time-dependent evolution towards the *α/β*-d-glucose equilibrium, a series of time interleaved measurements were made on the initial pure *α*-d-glucose solution. All even measurements were performed by selectively refocusing the 5.0–7.4 ppm range (i.e. the downfield spectrum), and all odd measurements by selectively refocusing the 1.8–4.2 ppm (i.e. upfield spectrum). The blue curve in Fig. 2a indicates the offset range where the RF pulse refocuses magnetization (for all values close to − 1.0). In the so-called stopband (for values close to 1.0) the pulse does not have a net effect. In the stopband, the transverse magnetization dephases further and no signal can be measured; this leads to additional lipid and water suppression. In the transition band (− 1.0 < value < 1.0) the RF pulse does not fully refocus the magnetization and partial refocusing occurs. With this RF pulse profile, the downfield spectrum was recorded. For the orange curve (Fig. 2a) the same applies; the only difference is that it is designed for optimal detection of the upfield spectrum. The following pulse sequences were used: TE = 120 ms, TR = 1500 ms, matrix = 65 × 23 × 7, FOV = 280 × 100 × 70 mm, resolution = 4.3 × 7.8 × 10 mm, displaced volume = 2.15 × 2.15 × 1 cm, averages = 1, and TA = 3:23 min.

#### In vivo α-d-glucose single-shot SLOW-EPSI (Scheme 1)

The CSAP pulse duration for in vivo measurement was 30 ms (orange curve in Fig. 1b). This sequence was applied to a healthy volunteer (Subject 1) at 7 T to evaluate the pulse sequence parameters and the detectable *α*-d-glucose in the human brain. Sequence parameters: TE = 80 ms, TR = 1500 ms, matrix = 65 × 23 × 7, FOV = 280 × 180 × 70 mm, resolution = 4.3 × 7.8 × 10 mm, averages = 6, and TA = 21 min.

#### In vivo tailoring asymmetric CSAP for optimal α-d-glucose detection (Scheme 2)

The asymmetric CSAP pulse^3^ with 20 ms pulse duration was designed for a shorter TE (60 ms) measurement (Fig. 3a,b). This sequence was applied to a healthy volunteer (Subject 2) at 7 T to evaluate the dynamics of *α*-d-glucose in the human brain. Sequence parameters: TE = 60 ms, TR = 1500 ms, matrix = 65 × 23 × 7, FOV = 280 × 180 × 70 mm, resolution = 4.3 × 7.8 × 10 mm, averages = 6, and TA = 21 min.

### Subjects

Subject 1: a healthy, nondiabetic, 30-year-old male; body weight, 45 kg. The measurement was performed without a fasting period. The measurements were performed using single-shot SLOW-EPSI (Scheme 1, TE = 80 ms).

Subject 2: a healthy, nondiabetic, 57-year-old male; body weight, 90 kg. The measurements were performed with asymmetric single-shot SLOW-EPSI (Scheme 2, TE = 60 ms) and a T_1_-weighted MRI according to the following protocol:

Experiment 1: (i) Subject 2 fasted for 13 h before the measurements; (ii) the blood sugar level was measured (5.6 mM) using a blood glucose monitor (GL44, Beurer Medical); (iii) the baseline SLOW-EPSI measurements were performed; (iv) the patient table of the scanner was moved back to the home position and the subject drank a fresh non-equilibrium *α*-d-glucose solution (70 g dissolved in 500 ml tap water which was prepared ~ 6 min before the start of the measurements) via a flexible polyethylene tube while lying on his back and remaining inside the head coil; (v) SLOW-EPSI MRSI measurements were continued, saving one complete SLOW-EPSI dataset every 3–4 min. The time schedule for these measurements is shown in Fig. 3c. (vi) the blood sugar level was measured again (5.7 mM) using a blood glucose monitor (GL44, Beurer Medical);

Experiment 2 The above-described procedure was repeated on a different day with the same subject. This time subject 2 drank an equilibrium *α/β*-d-glucose solution instead of *α*-d-glucose. The solution was prepared 650 min before the start of the measurements. The blood sugar levels were 4.9 mM and 4.8 mM before and after the measurements using a blood glucose monitor (GL44, Beurer Medical).

All experimental protocols were approved by the local ethics committee (Ethikkommission für die Forschung am Menschen, Kantonale Ethikkommission Bern (2019-00503)). Informed consent was obtained from the subjects.

### Data reconstruction and pre-post-processing

For the data reconstruction and pre-post-processing, the Metabolic Imaging Data Analysis System (MIDAS)^12^, and MATLAB R2019b were used. Further details are provided in the supplementary material.

### Quantification

Quantification of *α*-d-glucose was calculated using peak integration of 5.22 ppm and compared to the peak integration of internal acquired water signal at 4.65 ppm. The calculation is based on following assumptions: (i) water concentration = 55.5 mM; (ii) the water contents in grey and white matter are 75% and 65%, respectively; (iii) the Fraction of grey and white matter in the human brain are 40% and 60%, respectively; (iv) the T_1_ values at 7 T of water in grey and white matter are 2000 ms and 1357 ms, respectively; (v) the T_2_ values at 7 T of water both grey and white matter are 80 ms; (vi) the longitudinal magnetization *Mz* of water is zero after the first excitation pulse for metabolite acquisition (due to previous water suppression pulses); (vii) the T_1_ and T_2_ values of *α*-d-glucose at 5.22 ppm in the whole brain are 1780 ms and 121 ms (taken from literature on the T_1_ and T_2_ of total creatine at 7 T^13^), respectively.

## Supplementary Information

Supplementary Information.

## Data Availability

The MRS dataset used for this study is available for quality control from https://boris-portal.unibe.ch (https://doi.org/10.48620/111).

## References

[1] Gruetter R (1992). Direct measurement of brain glucose concentrations in humans by 13(C NMR spectroscopy (glucose transport/blood-brain barrier/in vivo NMR/glucose metabolism). Proc. Natl. Acad. Sci. U.S.A..

[2] De Feyter HM (2018). Deuterium metabolic imaging (DMI) for MRI-based 3D mapping of metabolism in vivo. Sci. Adv..

[3] Weng G (2022). SLOW: A novel spectral editing method for whole-brain MRSI at ultra high magnetic field. Magn. Reson. Med..

[4] Tkáč I (2021). Water and lipid suppression techniques for advanced ^1^ H MRS and MRSI of the human brain: Experts’ consensus recommendations. NMR Biomed..

[5] DeFronzo RA, Tobin JD, Andres R (1979). Glucose clamp technique: A method for quantifying insulin secretion and resistance. Am. J. Physiol..

[6] Joers JM (2017). Measurement of hypothalamic glucose under euglycemia and hyperglycemia by MRI at 3T. J. Magn. Reson. Imaging.

[7] Rivlin M, Navon G (2019). Molecular imaging of tumors by chemical exchange saturation transfer MRI of glucose analogs. Quant. Imaging Med. Surg..

[8] Van Zijl PCM, Yadav NN (2011). Chemical exchange saturation transfer (CEST): What is in a name and what isn’t?. Magn. Reson. Med..

[9] Slotboom J, Mehlkopf AF, Bovee WMMJ (1994). The effects of frequency-selective RF pulses on J-coupled spin-1/2 systems. J. Magn. Reson. A.

[10] Ebel A, Maudsley AA (2003). Improved spectral quality for 3D MR spectroscopic imaging using a high spatial resolution acquisition strategy. Magn. Reson. Imaging.

[11] Mescher M, Merkle H, Kirsch J, Garwood M, Gruetter R (1998). Simultaneous in vivo spectral editing and water suppression. NMR Biomed..

[12] Maudsley AA (2006). Comprehensive processing, display and analysis forin vivo MR spectroscopic imaging. NMR Biomed..

[13] Li Y (2013). T1 and T2 metabolite relaxation times in normal brain at 3T and 7T. J. Mol. Imaging Dyn..

