# Supplementary material for: α-D-Glucose as a non-radioactive MRS tracer for metabolic studies of the brain
Source: Sci Rep. 2023 Apr 15;13:6159. doi: 10.1038/s41598-023-33161-8 (PMC10105689; doi:10.1038/s41598-023-33161-8)
Supplement: Supplementary file 1 — Supplementary Information. [file 41598_2023_33161_MOESM1_ESM.docx]

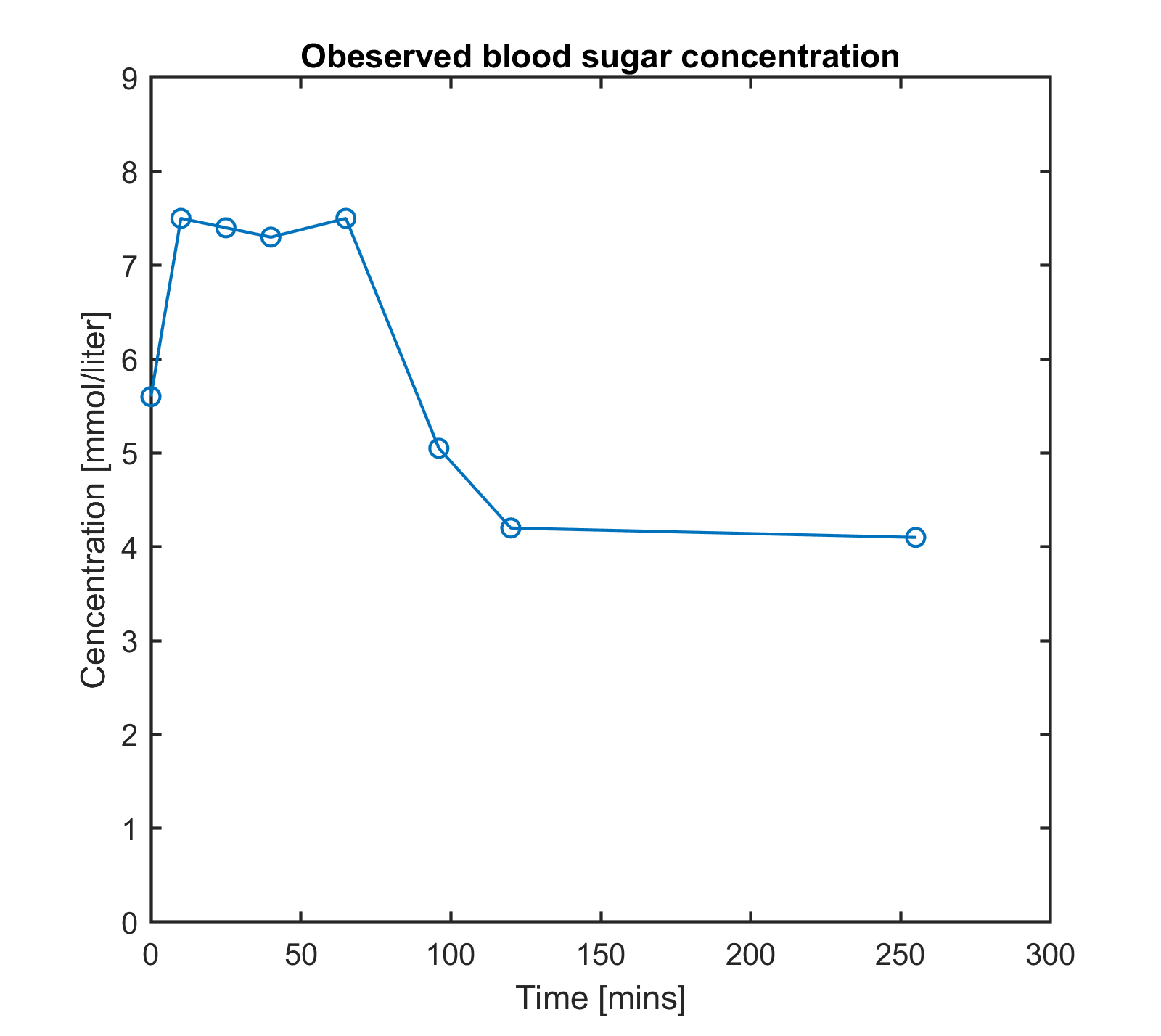


**Figure S1: Measured blood sugar levels** in subject 2 in a separate experiment on a different day without MRSI measurements. Test subject fasted for more than 12 hours before the experiment, and ingested 65.8 gram of α-D-glucose dissolved in 500ml cold tap water. The blood glucose level was 5.6 mmol/liter when glucose was ingested, and increased by ~34% after 10 mins (~7.5 mmol/liter), and decreased to 4.2 mmol/liter after 120 mins.

## Reconstruction and pre-post-processing

The flowchart of reconstruction and pre-post-processing is illustrated on Supporting Information Figure S2.

*Raw data*

The measured raw-data is reconstructed using ICE and stored into DICOM format, which is further processed by Metabolic Imaging Data Analysis System (MIDAS) [1].

*MIDAS*

MIDAS is a software-program written in IDL™ (Harris Geospatial Solutions, Inc., Colorado, USA) used to process EPSI-data. The processing-pipeline is the following:

1. Non-uniform EPSI readout k-space regridding is accomplished using interlaced Fourier-transform [2].
2. The even and odd echoes are averaged with echo-drift-correction.
3. Spatial Fourier-transforms (FFT) with Gaussian filter for water reference data.
4. Linear regression was performed for FID points 3 and 7 to create phase and magnitude correction functions using water reference data. The reason for this is to avoid oscillations in the 1st few echoes. Then a weighted combination was performed for multi-channels [3]. The phase and magnitude correction and weighting of water reference data were saved and then used in the subsequent spatial FFT of metabolite data.
5. Eddy current correction.
6. Spectral FFT-with Gaussian-filter (with a broadening-parameter in the range of 2 - 6 ).

*MATLAB*

1. Automatic phase-correction and frequency-shift correction was applied based on the reference water signal at 4.65 ppm.
2. Volume-of-interest selection.
3. (Optional) Apodization-filter in time-domain.
4. (Optional) Filter out voxels with poor spectral quality based on lipid contamination signal.
5. (Optional) Baseline-correction, lipid contamination and residual water removal using splines.


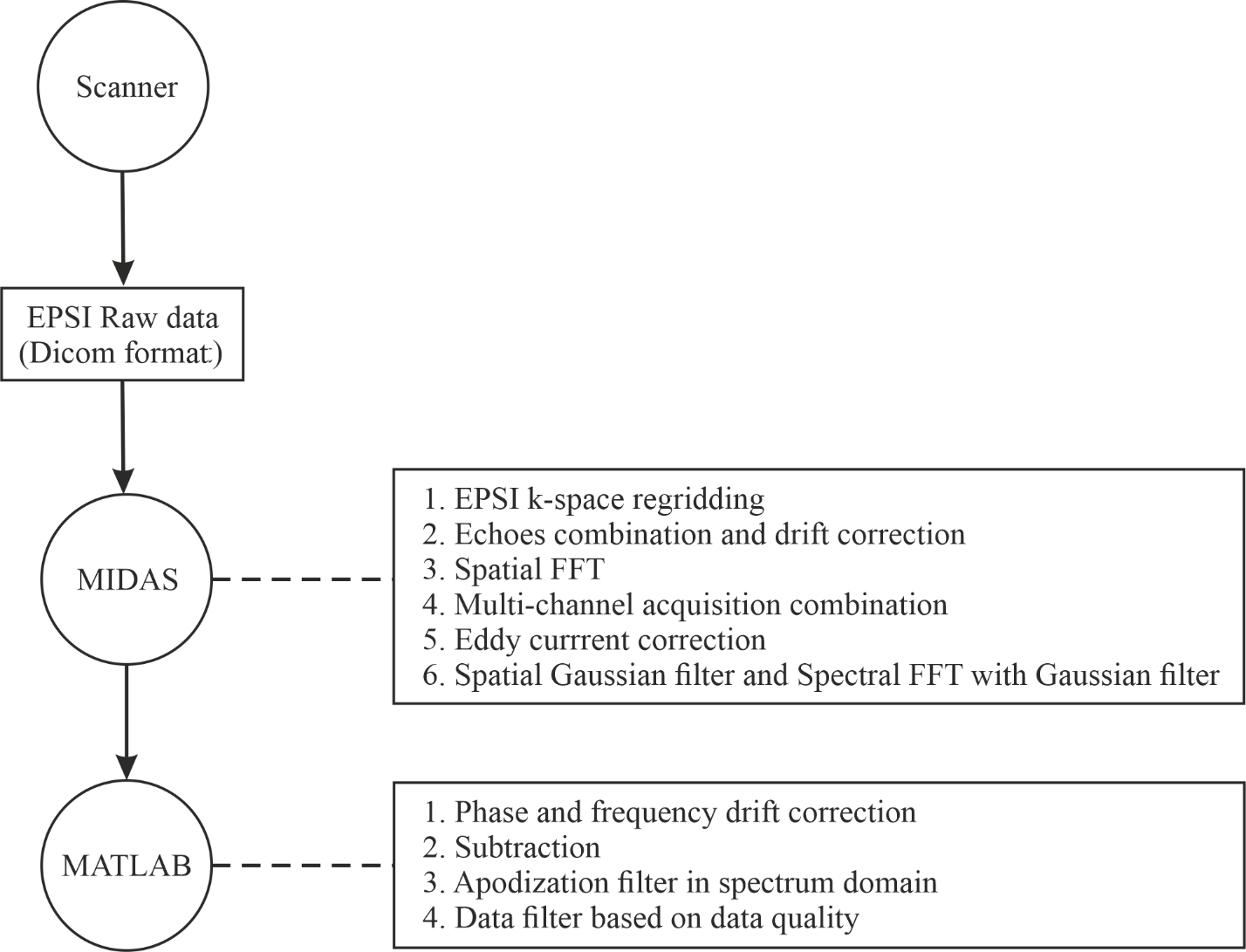


**Figure S2:** Flowchart of reconstruction and pre-post-processing.

## References

[1] A. A. Maudsley *et al.*, “Comprehensive processing, display and analysis forin vivo MR spectroscopic imaging,” *NMR in Biomedicine*, vol. 19, no. 4, pp. 492–503, Jun. 2006, doi: 10.1002/nbm.1025.

[2] H. Bruder, H. Fischer, H.-E. Reinfelder, and F. Schmitt, “Image reconstruction for echo planar imaging with nonequidistantk-space sampling,” *Magnetic Resonance in Medicine*, vol. 23, no. 2, Feb. 1992, doi: 10.1002/mrm.1910230211.

[3] A. Abdoli and A. A. Maudsley, “Phased-array combination for MR spectroscopic imaging using a water reference,” *Magn Reson Med*, vol. 76, no. 3, pp. 733–741, Sep. 2016, doi: 10.1002/MRM.25992.
